# Identifying relevant concepts and factors for the sustainability of evidence-based practices within acute care contexts: a systematic review and theory analysis of selected sustainability frameworks

**DOI:** 10.1186/s13012-019-0952-9

**Published:** 2019-12-19

**Authors:** Letitia Nadalin Penno, Barbara Davies, Ian D. Graham, Chantal Backman, Ibo MacDonald, Julie Bain, Alekhya Mascarenhas Johnson, Julia Moore, Janet Squires

**Affiliations:** 1 School of Nursing, Faculty of Health Sciences, University of Ottawa, 451 Smyth Road, Ottawa, ON K1H 8M5 Canada; 2 School of Epidemiology and Public Health, University of Ottawa, 600 Peter Morand, Crescent, Ottawa, ON K1G 5Z3 Canada; 3 School of Medicine, University of Ottawa, 451 Smyth Road, Ottawa, ON K1H 8M5 Canada; 4 Sunnybrook Health Sciences Centre, Regional Geriatric Program of Toronto, 2075 Bayview Avenue, Toronto, ON M4N 3M5 Canada; 5 The Center for Implementation, 20 Northampton Dr, Toronto, ON M9B 4S6 Canada; 6 Ottawa Hospital Research Institute, University of Ottawa, 451 Smyth Road, Ottawa, ON K1H 8M5 Canada

**Keywords:** Frameworks, Models, Theories; Sustainability, Sustainment, Routinization, Institutionalization, Utilization, Evidence-based practices/guidelines/programs/interventions, Innovations

## Abstract

**Background:**

There is growing recognition among healthcare professionals that the sustainability of evidence-based practices (EBPs) within different settings is variable and suboptimal. Understanding why a particular EBP might be sustained in one setting and not another remains unclear. Recent reviews illustrate the need to identify and analyze existing frameworks/models/theories (F/M/Ts) that focus solely on the sustainability of EBPs in specific healthcare settings, such as acute care, to illuminate key determinants and facilitate appropriate selection to guide practice and research.

**Methods:**

We conducted a systematic review to extract sustainability frameworks. This involved using two available syntheses of the literature and a systematic search of four databases from January 2015 to July 2018: CINHAL, MEDLINE, Embase, and ProQuest. We included studies published in English, and if they included sustainability F/M/Ts recommended for use in acute care or an unspecified healthcare organization/setting. F/M/Ts explicitly recommended for use in public health and or community settings were excluded. We then conducted a comparative analysis of F/M/Ts using a modified theory analysis approach, to understand the theoretical underpinnings of each F/M/T, their determinants and concepts hypothesized to influence the sustained use of EBPs within an acute care context.

**Results:**

Of 2967 identified citations from the 2 available syntheses and the systematic review, 8 F/M/Ts met the inclusion criteria. We identified 37 core factors, of which 16 were recorded as common factors (occurring within 4 or more of the 8 included F/M/Ts). All factors grouped into 7 main themes: innovation, adopters, leadership and management, inner context, inner processes, outer context, and outcomes.

**Conclusions:**

This systematic review is the first to include a comprehensive analysis of healthcare sustainability F/M/Ts for the sustained use of EBPs in acute care settings. Findings reveal insights into sustainability as a “process or ongoing stage of use” following initial implementation, suggesting this construct should be added to the definition of sustainability. Results provide a resource of available F/M/Ts and hypothesized factors to consider for acute care team members who are planning or currently implementing EBPs with the goal of improving patient outcomes. It also provides a basis for future research on sustainability in acute care.

Contributions to the literature

- This review identifies 8 sustainability frameworks/models/theories (F/M/Ts), 7 key themes/constructs and 37 factors hypothesized to influence sustained use of evidence-based practices (EBPs) for acute care team members who are planning or currently implementing EBPs with the goal of improving patient outcomes.
- Of the 7 themes/constructs identified for acute care, 4 align with current literature, and 3 add to the body of evidence.
- The analysis provides insight into sustainability as a process or ongoing stage adding to the current definition for sustainability.
- The modified theory analysis tool can be used to examine concepts and factors of emerging or existing F/M/Ts.

## Background

Over a decade ago, the sustained use of evidence-based practices (EBPs) was identified as a gap in the literature. Evolving debate among experts suggest sustainability should be considered a distinct concept that occurs “(1) after a defined period of time, (2) the program, clinical intervention and/or implementation strategies (hereafter referred to as EBPs) continue to be delivered and/or, (3) individual behavior change (i.e. clinician, patient ) is maintained, (4) the program (EBP) and individual behavior change may evolve or adapt while (5) continuing to produce benefits for individuals/systems [1]”. Despite growing interest, the timing and understanding of how to sustain the use of EBPs remains a relatively unexplored field of research [2, 3] and least understood part of the translation research process [4] that has challenged practitioners and researchers alike. Evidence reveals the integration and sustainability of EBPs in clinical practice is “an iterative, dynamic” [5] and “complex process,” [6] which poses a significant challenge. Emerging discourse indicates efforts to sustain EBPs in healthcare should be guided by conceptual frameworks, models or theories (hereafter collectively referred to as F/M/Ts) [1, 7–12] to better understand the factors that impact sustainability as a distinct concept [13, 14], over time, in a range of distinct healthcare settings [3, 10, 11]. Thus, a critical analysis of existing sustainability F/M/Ts relevant to acute care contexts was conducted as a way to understand the meaning of key concepts, factors, and their relationships to ultimately provide direction for practice and research.

Increasing demand on healthcare organizations to improve patient outcomes [10, 15, 16] in an efficient, cost-effective manner [17, 18]) has resulted in the growing expectation that EBPs be informed by research, be effective and sustainable to inform clinical decision making [19, 20]. In response, healthcare organizations have undertaken a number of quality improvement initiatives [10]. Despite efforts, variable rates of sustained use of EBPs exist ranging from none to full adherence [2], not only among various healthcare professionals but also within different settings [1, 9, 10, 17, 20–24]. Researchers argue the decay of sustained EBPs [17, 23, 25, 26], also referred to as the “improvement evaporation effect” [25, 26] can be attributed to the limited use of theoretical F/M/Ts [27, 28]. To overcome these challenges and to advance knowledge, researchers [7, 11–14] recommend the use of F/M/Ts to examine the factors that impact sustainability as a distinct concept, especially in complex acute care environments [11].

Recent reviews/syntheses reveal a lack of use/empirical testing of existing F/M/Ts [10, 19, 24], highlight several diverse perspectives, applications and constructs deemed useful for sustainability [10], and few F/M/Ts that focus solely on the sustainability of EBPs within acute settings [10, 24, 29]. Specifically, the majority of sustainability F/MTs and approaches are designed for use in non-specified healthcare settings (37% or 23/62) (e.g., healthcare organizations or systems), followed by 31% (19/62) specified for use in public health, 26% (16/62) in community settings, and only 3% (2/62) primarily focused within acute care [10]. To date, a review that examines how to improve the sustainability of EBPs in acute care settings has not been conducted [11]. Given healthcare expenditures are reported to be the largest in hospitals (36.9% in 2018) [30], exclusively identifying relevant concepts and factors related to sustainability in this challenging setting will likely be of considerable benefit to research and practice, potentially improving the quality of care and reducing costs. Clearly, a gap exists regarding which existing sustainability F/M/Ts are applicable and what factors are relevant when trying to sustain the use of EBPs primarily in acute care contexts [10, 13].

The aims of this study were to (i) identify existing healthcare F/M/Ts that explicitly address the process of sustained use of research (EBPs/guidelines/innovations/clinical protocols/programs/interventions) and are recommended for use within acute care contexts or unspecified healthcare organization/setting; (ii) compare F/M/Ts, using a theory analysis approach, to identify key concepts and factors that influence/predict the likelihood of successful sustainability of EBPs; and (iii) provide a list of relevant sustainability F/M/Ts, concepts, and core factors to act as a guide for practice and provide direction for future research within acute care contexts.

## Methods

### Search strategy and data sources

Two different data sources and related search strategies were used to identify existing healthcare sustainability F/M/Ts. First, a full text review, abstraction and appraisal of all F/M/Ts included in two existing knowledge syntheses of sustainability in healthcare [1, 10] was conducted to determine overall alignment with the aims and eligibility criteria established for this study given their original purposes, scopes and related methodologies (see Table 1). Second, to identify relevant sustainability F/M/Ts published after the two syntheses, a new systematic search of all published articles, dissertations/theses, systematic and scoping reviews, and concept analyses was conducted using the same eligibility criteria guided by the Preferred Reporting Items for Systematic Reviews and Meta-Analysis (PRISMA) reporting standards [31, 32] (see Additional file 1). The selection of healthcare databases, search terms, and strategy was supported by a health science librarian and peer-reviewed by a second using the PRESS EBC Checklist [33]. A search of CINHAL, MEDLINE, Embase, and ProQuest databases was conducted with results limited to citations published between 1 January 2015 and 3 July 2018, based on end dates of the two syntheses. A hand search of references from included citations was undertaken. Details of key terms and search strategies are available in Additional file 2. Ethical approval was not required for this review.

**Table 1:** Inclusion and exclusion criteria for systematic review (January 1, 2015, to July 3, 2018)

Inclusion criteria	
Study Design	All published articles and dissertations/theses, systematic and scoping reviews, concept analysis
Publication Dates	Published between January 2015 and July 3, 2018. Based on reviews by Moore et al. (2017); and Lennox et al. (2018) Represents the most refined version of the framework/model/theory (F/M/T)
Setting	Recommended for use in acute care setting Recommended for use in any healthcare organization in general and did not specify a specific healthcare setting. Must explicitly provide factors and concepts relate to sustainability
Outcomes	Primary outcome: A sustainability F/M/T that addresses the process of sustained use of research (evidence-based practice/guidelines/innovation/clinical protocol/programs/interventions) Provides a definition of sustainability. Because sustainability is defined numerous ways, we included all studies in which the originators used one of the following terms sustainability, routinization, institutionalization. Provides information on the theoretical underpinnings and evidence supporting the F/M/T Provides information on the concepts and related factors influencing sustainability of evidence-based practice/guidelines/innovation/clinical protocol/programs/interventions.
Exclusion criteria	
Publications	Exclude if not a unique and index version (most up to date) of the F/M/T
Setting	Exclude if not recommended for use or applicable within a healthcare organizational practice setting Exclude if not explicitly recommended for use within acute care or unspecified healthcare organization/setting Exclude if explicitly recommended for use in a specific setting such as public health or community setting, or has a health promotion focus
Language	Exclude all citations in any other languages than English
Outcomes	Exclude if no F/M/T is included Excluded if about delivery system components and no F/M/T model included Exclude if only describes factors related to sustainability and no F/M/T is included Exclude if it contains both initial implementation and sustainability and does not explicitly provide a detailed breakdown of related sustainability concepts and factors. Excluded if the F/M/T being described is not about healthcare innovations/evidence-based practices

### Eligibility criteria

Eligibility criteria were designed to examine sustainability as a distinct concept, as per Moore et al.’s [1] definition, and to identify concepts and factors that related solely to the sustained use of EBPs, after the initial rollout, in complex healthcare environments such as acute care [3, 34]. A checklist of inclusion and exclusion criteria was developed to guide selection of citations (see Table 1). During the process, four coauthors (LNP, JS, BD, CB) reviewed a sub-sample of citations [25] to refine and ensure criteria could be consistently applied. To be eligible, citations needed to be published in English; in a peer-reviewed journal; include sustainability or implementation and sustainability F/M/Ts recommended for use in acute care or an unspecified healthcare organization/setting; and represent the most current/refined version.

A citation was excluded if the F/M/T was not recommended for healthcare; was recommended only for use within a specified setting other than acute care (e.g., public health or community); if it contained only an implementation F/M/T; and if it contained an implementation and sustainability F/M/T without an explicit breakdown of related sustainability factors. Notably, this study was not designed to examine the influence of implementation on sustainability.

### Data collection process and analysis

A data collection form was piloted by four coauthors (LNP, BD, CB, JS) with 50 randomly selected citations to ensure comprehensiveness prior to screening. The form required minimal modification. To ensure inclusiveness, level 2 full text screening of all citations was conducted in two steps: (i) screening of results from two syntheses was completed by one reviewer (LNP) and reviewed by four coauthors (BD, IG, CB, JS); (ii) screening of systematic review results was completed by two independent reviewers (LNP, IM). Final decisions regarding inclusion were made jointly by LNP and coauthors (BD, IG, CB, JS). Disagreements were resolved through discussion and consensus.

A theory analysis of the identified F/M/Ts was undertaken as a means of understanding their theoretical underpinnings, paying particular attention to key concepts/factors influencing sustained EBP use within acute care [35]. According to Walker and Avant [36], theory analysis involves consideration of seven elements: (i) determining origins, (ii) examining meaning of concepts and their relationships; (iii) analyzing logical adequacy of concepts and relational statements to determine predictive ability to generate hypotheses, (iv) determining usefulness for practice and predicting outcomes, (v) defining generalizability across settings, (vi) defining the degree of parsimony and language clarity, and (vii) determining testability (see Table 2). Modifications to the theory analysis elements/tool included adding a subjective rating scale for both parsimonious (full or partial) and language (clear, somewhat unclear, unclear). Analysis involved entering findings into a master chart to facilitate comparisons. All factors identified in the appraisal were then extracted and collated. Qualitative content analysis was completed by identifying and placing all related and similar factors together (identified as *core factors*) and then into broad *themes*, which were inductively identified from F/M/Ts [37, 38]. Factors cited in four or more F/M/Ts within each theme were identified as *common factors*.

**Table 2:** Theory analysis elements applied to sustainability frameworks/models/theories

Categories	Criteria
Origins	Who are the developers, discipline, country? Methodological approach Evidence to support or refute model development Target domain (practice, education, research, policy) Motivation(s) for development
Meaning of the framework/model/theory (F/M/T)	Examines conceptual definitions and their use Identifies concepts (factors), Inclusiveness of innovation, potential adopters, context factors Relationship between and among concepts (factors) Assumptions underlying the model (preconditions) Schematic presentation
Empirical testability	Supported by empirical data (studies)
Parsimonious Language	Clarity and simplicity while being complete (as per rater) Use of clear, concise language (as per rater)
Logical adequacy	Logical adequacy (logical structure of the concepts and statements) Predictions or testable hypotheses are provided Logical fallacies within the content or structure of the model
Usefulness	Supported by tools Practicality to nursing and or other target groups. Contributes to the understanding and predicting of outcomes
Generalizability	Clinical context, generalizes (can be extended) to multiple settings

Based on Walker and Avant [36]

## Results

**Fig. 1:**
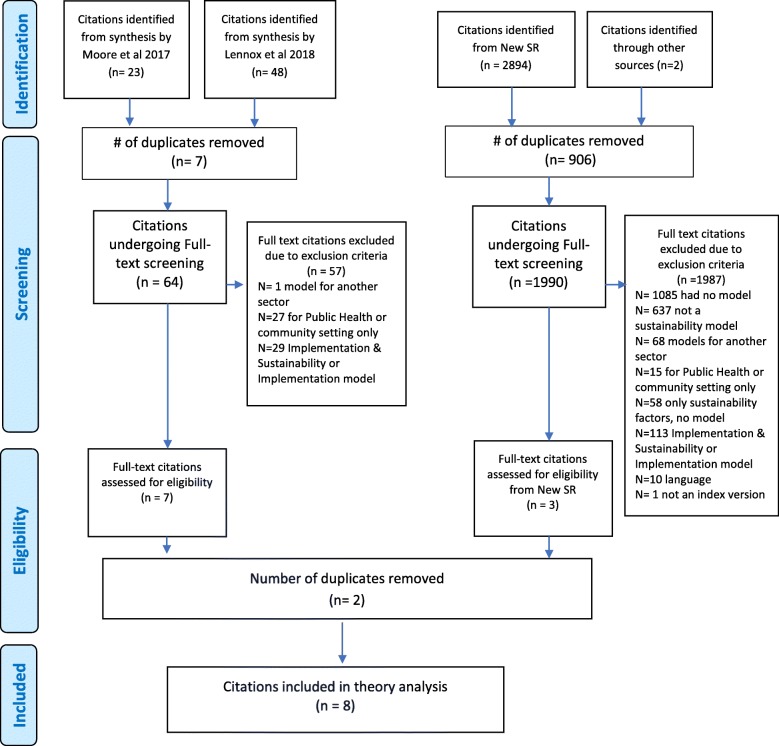
PRISMA flow diagram for combined syntheses (Moher et al. 2009)

Of the 2967 citations identified, 8 met the inclusion criteria (e.g., four from Moore et al. [1], three from Lennox et al. [10], one from the new systematic review) and were eligible for theory analysis (Fig. 1). Rationale for excluded citations is documented in Additional file 3*.* Most F/M/Ts containing both implementation and sustainability phases did not explicitly provide a detailed breakdown of the sustainability concepts/factors and were excluded. Those that did were recommended for use in community and/or public health settings and were excluded.

### Framework/model/theory characteristics and quality appraisal

#### Origins

The F/M/Ts were published between 2005 and 2016; the majority (*n* = 6) published after 2010. The originators are from Europe (*n* = 4) [25, 39] [40, 41]; North America (*n* = 3) [13, 34, 42]; and Australia (*n* = 1) [43] (see Table 3). Various methodological approaches used by originators to develop F/M/Ts included focused systematic or literature reviews (*n* = 4) [25, 34, 40, 42]; integrative reviews of frameworks or theory [41, 43]; a Bayesian research co-production approach [39]; and a concept analysis [13]. The F/M/Ts were reported to be based on theoretical and empirical work of scholars from different fields of study/disciplines with varying theoretical perspectives on sustainability. Specifically, these included the diffusion of innovations theory [42, 43], organizational change theory [25], organizational and management theory [25, 39, 42], ecological theory [34, 43], total quality improvement theory [25], psychological theory [41], theory of routines [40], and multiple healthcare discipline theories [13]. Six F/M/Ts were designed to be operationalized to guide practice and/or research at an organizational or unit/departmental level. Two were specified for use at the project/initiative level [34, 39]. Three F/M/Ts were explicitly recommended for use in a hospital [13, 40, 41], and the remaining five were recommended for use in any unspecified healthcare organization/setting. Some F/M/Ts were intended for multiple audiences, namely, researchers [13, 25, 34, 39–42] practitioners [13, 34, 39–42], policy-makers [34], administrators [13], and funders/grantors [42]. The most common motivation was to add to the body of evidence/knowledge to either guide research or better understand how to successfully sustain effective improvements in practice.

**Table 3:** Origins of sustainability frameworks/models/theories for acute care settings

First author	Year	Country of origin	Name of F/M/Tl	Methodological approach used	Basis or field of study derived from	Recommended setting for use	Context level	Target audience
Buchanan et al. [25]	2005	UK	Sustaining Organizational Change Framework (SOCF)	Focused Systematic Review	-Organizational (orgal) change theory -Management (Mgmt.) and total Quality Improvement (QI) theory	Recommended for different types of change and different contexts—organizational settings providing health and human care services (p. 189)	Unit or organizational level	Researchers concerned with organizational change (p. 190)
Racine [42]	2006	USA	Model for Sustaining Innovations in their effectiveness (MSI)	Focused Systematic Review	-Diffusion of Innovation theory -Orgal and Mgmt. theory	For use in health and human service innovations and related contexts, (p. 357, 381)	Unit or organizational level	Blueprint (p. 382) for funders, grantors, researchers and practitioners (p. 356-7)
Maher et al. [39]	2010	UK	NHS Sustainability Model (NHS SM)	Bayesian subjective research co-production approach to identify and rank factors	-Orgal and Mgmt. theory	Healthcare settings and service innovations (p. 5 of guide)	Project or initiative level	Inter-disciplinary researchers and practitioners (p. 5)
Slaghuis et al. [40]	2011	Netherlands	A Framework and a Measurement Instrument for Sustainability of Work Practice in long term care (FMIS WP)	Literature review of the concepts “routinization” and “institutionalization”	-Theory of routines	Applicable to multiple settings and service organizations in and out of healthcare including hospital care, long-term care (p. 323)	Department or organizational level	Researchers and practitioners seeking to measure if changed practices are sustained (p. 314)
Chambers et al. [34]	2013	USA	Dynamic Sustainability Framework (DSF)	Literature review of the concepts “voltage drop” and “program drift”	-Ecological theory	Recommended for a broad range of healthcare service interventions and a myriad of clinical organization and community settings (p. 125)	Project or initiative level	Researchers, policy-makers, practitioners (p. 117, 123-4)
Fox et al. [43]	2015	Australia	Sustainability of Innovation Theoretical Framework (SITF)	A synthesis of theoretical propositions from an integrative review featuring 2 frameworks: Greenhalgh et al 2004 and Chambers et al. 2013	-Diffusion of Innovation theory	Broad range of healthcare service innovation in several contexts including nursing contexts (p. 73)	Unit or organizational level	Researchers (p. 70 ,74)
Fleiszer et al. [13]	2015 and 2016	Canada	Sustainability of Healthcare Innovations Framework (SHIF)	Concept analysis of ‘innovation sustainability”	-Theories from multiple disciplines (health, social services, public healthy, mgmt.)	Diverse frontline acute healthcare nursing settings (2016, p. 215)	Unit level	Inter-disciplinary researchers, practitioners, administrators (p. 1484–5)
Frykman et al. [41]	2017	Sweden	DCOM Framework with Realistic Evaluation (DCOMF)	Integrative review combining an organizational framework grounded in psychological theory with Realistic Evaluation	-Psychological theory of applied behavior analysis	Complex changing healthcare context such as emergency depts (p. 76)	Organizational level	Researchers and inter-professional practitioners (p. 64, 76)

#### Meaning of the F/M/T

To examine how originators defined the constructs of sustainability, conceptual definitions for sustainability were mapped to the five constructs of a comprehensive definition recently published by Moore et al. [1] (see Table 4). Notably, one author did not provide nor reference an explicit definition of sustainability [42]. Two definitions included all five established constructs [25, 39], and four definitions included all but one construct [13, 34, 40, 41]. Similar to Moore et al. [1] findings, the most commonly described construct for sustainability was “continued delivery or use,” which was combined equally with the “evolution or adaption” construct cited in seven out of eight definitions.

**Table 4:** Framework/model/theory sustainability definitions mapped to sustainability constructs by Moore et al. [1]

Reference	Synonym	Definition	Sustainability constructs by Moore et al [1]					No. of constructs total = 5	New constructs	
			After a period of time	Continued delivery or use of innovation	Maintain behavior change (use of innovation) by individuals	Evolution or adaptions of innovation	Continued benefits of using innovation		Defined as process*	Defined as a stage**
Buchanan et al. [25]	Sustainability	The sustainability of change can be broadly defined as the 'process' through which new working methods, performance goals and improvement trajectories are maintained for a period appropriate to a given context [25].	x		x		x	*3*	x	
Racine [42]	Sustainability	None provided The framework alludes to the existence of 'stages' in the process through which an innovation goes from adoption to sustainability						0		*x*
Maher et al. [39]	Sustainability	Sustainability is when new ways of working and improved outcomes become the norm. Not only have the 'process' and outcome changed, but the thinking and attitudes behind them are fundamentally altered and the systems surrounding them are transformed in support. In other words, it has become an integrated or mainstream way of working rather than something “added on.” As a result, when you look at the process or outcome one year from now or longer, you can see that at a minimum it has not reverted to the old way or old level of performance. Further, it has been able to withstand challenge and variation; it has evolved alongside other changes in the context, and perhaps has actually continued to improve over time [40].	x		x	X	X	4	x	
Slaghuis et al. [40]	Routinization	Sustainability is “a dynamic 'process' in which actors in a targeted work practice develop and/or adapt the organizational routines to a new work method. This process can also be described as routinization: through the development of organizational routines a new work method becomes part of everyday routine activities. This process also involves learning processes at different levels in the organization, as there is more to the daily performance of a work practice than just routinization” (40).		x	x	x		*3*	x	
Chambers et al. (34)	Sustainability	Sustainability is a 'process' of managing and supporting the evolution of an intervention within a changing context [35]. Sustainability has evolved from being considered as the endgame of a translational research process to a suggested “adaptation phase” that integrates and institutionalizes interventions within local organizational and cultural contexts (34].	X	x		x	x	4	x	
Fox et al. [43]	Sustainability	No explicit definition is provided with framework. ...only explicitly states it combines the concepts presented by Greenhalgh et al. [44] in their systematic review (e.g., successful routinization is strongly impacted by staff continuity, attrition, and perceptions of the value and need of the innovation), and the DSF Chambers et al. [34] which posits the concept is not an endpoint but rather involves a 'process' of innovation evolution or continual adaptation as a result of learning, problem-solving and evolution [43].	x	x	x	x		*4*	x	
Fleiszer et al. [13]	Sustainability	Sustainability is a 'process' that emerges from and succeeds innovation implementation wherein improvements are maintained, new ways of working become routine, surrounding systems are transformed in support and the innovation may even be developed, over a period of time appropriate to a given situation [13].	x	x		x	x	4	x	
Frykman et al. [41]	Sustainability	Uses Stirman et al. [9] definition of sustainability, “the 'phase' of implementation when initial support has been withdrawn, core elements are maintained, and capacity for continued performance of the core elements is maintained” [41].	x	x		x	x	4		*x*
Total definitions referencing the construct			6	5	4	6	5		6	2

*Defines sustainability as a process [25], [39], [40], [34], [43], [13]

**Defines sustainability as an ongoing stage or phase of implementation [42], [41]

Differing from Moore et al. [1] findings where most publications did not define the timeline for sustainability, the “after a period of time” construct was included in 75% (6 out of 8) of the definitions. However, these time-related references were undefined and unquantified. The “continued benefits” construct occurred in five out of eight definitions signifying the importance of the perceived goal to enhance outcomes (on individual, unit, organization, system level). The “maintain behavior change in individuals” construct reflected how a broad range of EBPs may interact with individuals or teams to maintain behavior change for sustainability. Although this was the least commonly described construct, it occurred in half the definitions: two F/M/Ts recommended for use in acute care [40, 41] and two for use in unspecified settings [25, 39].

Two similar constructs of sustainability currently not included in the Moore et al. [1] definition emerged during the analysis: defining sustainability as a “process,” [13, 25, 34, 39, 40, 43] or as a “stage/phase of ongoing use” post-implementation [41, 42]. These views were supported by several theoretical perspectives given F/M/T origins, revealing a new construct that describes the nature of sustainability to be “ongoing/continuous and process-like.”

#### Synthesis of factors and themes

Initially, 152 sustainability factors were extracted from the 8 F/M/Ts. Qualitative analysis identified 37 *core factors*, which grouped into 7 *themes*: (1) characteristics of the *innovation***/**EBP; (2) *adopter/user* factors influencing sustained use (3) *leadership and management* influences/factors; (4) *inner context* (practice setting/organization) factors where EBPs are delivered; (5) *inner processes***/**infrastructure factors that support the EBPs (e.g., processes, methods, systems, structures, or strategies); (6) *outer context* or broader system factors; and (7) *outcomes* descriptions without defined factors. Further synthesis identified *16 common factors* (occurring in four or more F/M/Ts), which are highlighted with an asterisk in Table 5.

**Table 5:** Synthesis of themes and factors found in sustainability frameworks/models/theories for acute care (*n* = 8)

Theme/concept	Core factors	Unspecified setting Fwks					Acute care Fwks		
		*1*	*2*	*3*	*5*	*6*	4	*7*	*8*
Innovation (defined as: new process/change/product/practice or program, innovation, intervention)	*Relevance/consistent with competitive strategy	✓	✓			✓		✓	
	*Characteristics (scale, shape and form, age, nature, type, integrity)	✓	✓		✓			✓	
	*Perceived centrality to organizational performance/platform/services	✓	✓		✓			✓	
	Fit with org’s vision/mission, procedures/strategies	✓		✓				✓	
	Adaptability of innovation			✓		✓		✓	
	*Benefits to patient, staff, organization (cost-effective, efficiency and quality of care)		✓	✓	✓	✓		✓	
	Barrier identification					✓			
Adopters (defined as staff, stakeholder, user, adopter, actor, and or individual)	Human resources - recruitment, processes, succession and leave planning (staffing)				✓	✓			
	*Individual commitment to innovation	✓	✓			✓		✓	
	*Individual competency (skill knowledge, absorptive capacity) to perform innovation	✓	✓		✓			✓	✓
	Internal cohesion between individual and commitment within the organization/stakeholder engagement leads to increased performance		✓					✓	✓
	Stakeholder commitment to innovation			✓			✓		✓
	Stakeholder beliefs, attitude, perceptions, emotions, expectations towards innovation	✓		✓		✓			
	Champion presence and involvement					✓		✓	
Leadership and management (defined as style, approach, behaviors, engagement support, or feedback)	*Management approach and engagement	✓	✓	✓	✓			✓	✓
	*Senior leadership involvement and actions	✓	✓	✓				✓	
Inner context (defined as context, practice setting or organization)	*Infrastructure support—policies and procedures based on innovation	✓		✓				✓	✓
	Infrastructure support for innovation in the job description with the mechanism for recognizing achievement	✓		✓			✓		
	*Infrastructure support-equipment and supplies for innovation			✓			✓	✓	✓
	Organization—absorptive capacity for innovation							✓	✓
	Cultural—beliefs, values, and perceptions to innov	✓						✓	
	*Cultural—climate	✓	✓		✓			✓	
	Cultural—innovation integrated into Norms (documents, protocols, manuals)	✓					✓		
	Political internal stakeholder coalition, power, influence	✓				✓		✓	
	Financial performance budgeting and measurement	✓				✓			
	Financial-internal funds and other non-financial resources of innovation					✓		✓	
Inner processes (defined as processes, methods, systems, structures, or strategies)	*Education and training processes			✓	✓	✓	✓	✓	
	Processual—planning, method, and timing of embedding innovation	✓					✓	✓	
	*Processual—project structure and system to monitor/manage innovation	✓		✓	✓		✓	✓	
	*Organization—communication capacity for monitoring (exchange and feedback)	✓	✓	✓	✓	✓	✓	✓	
	Behavioral change strategies								✓
Outer context (defined as external condition, context, system, or environment)	Socio-economic political threats, stability	✓			✓			✓	
	*External conditions, compatibility for innovation	✓	✓		✓			✓	
	Connection to broader external context		✓			✓		✓	
	External support for innovation from stakeholders	✓	✓					✓	
	*Political—policy, legislation, and interests		✓		✓	✓		✓	
	Financial-internal funds and other non-financial resources of innovation							✓	
Outcomes (defined as outcomes, teamwork behaviors, consequences, or continuation of benefits)	No factors explicitly defined in frameworks for this concept	✓				✓		✓	✓

*1* = Buchanan SOCF, *2* = Racine MSI, *3* = Maher NHS-SM, *4* = Slaghuis FMIS-WP, *5* = Chambers DSF, *6* = Fox SITF, *7* = Fleiszer SIHF, *8* = Frykman DCOMF

*Common factors**—**occurs in 4 or more frameworks

A subgroup analysis comparing the themes and factors among the specified acute care F/M/Ts [13, 40, 41] with those recommended for unspecified healthcare settings [25, 34, 39, 42, 43] was conducted. Results are available in Additional file 4 and Table 5. Notably, originators collectively identified all seven themes within both subgroups. Only three out of 37 core factors were uniquely identified among all F/M/Ts: two core factors were separately identified in two different F/M/Ts within the acute care subgroup (e.g., behavioral change strategies [41], financial funds, and non-financial resources [13]), and one core factor was identified within the unspecified setting subgroup (e.g., barrier identification [43]). Given minimal subgroup differences, all F/M/Ts were included in the theory analysis.

The themes were defined by terms used by originators. The “adopter” theme is defined as a stakeholder, staff, user, adopter, actor, or individual using the innovation/EBP. Of note, the *Sustainability of Innovation Theoretical Framework* (hereafter Fox SITF) [43] and *Sustainability of Healthcare Innovations Framework* (hereafter Fleiszer SHIF) [13] focused exclusively on the presence and influence of champions. The “inner context” theme refers to the context, practice setting or organization, while the “inner process” theme includes processes, methods, systems, structures or strategies used within the context. The “innovation” theme, defined as a new process, change, product, practice, or programme in six F/M/Ts, is not evident in two F/M/Ts [40, 41]. Similarly, the “leadership and management” theme refers to leadership style, approach, behaviors, engagement, support, or feedback in six F/M/Ts [13, 25, 34, 39, 41, 42]. The “outer context” theme, referencing conditions, context, systems or environment external to the inner context, is not evident in three F/M/Ts [39–41]. The *outcome* theme is described in four F/M/Ts as “outcomes on a spectrum from high to nil” [13], sustained “teamwork behaviors” [41], “consequences” [25], or “continuation of benefits” [34].

#### Inclusiveness of themes and factors

Three F/M/Ts [13, 25, 34] contain all seven themes with one F/M/T [42] containing six themes. The inclusiveness of 6–7 themes in 50% (4 out of 8) F/M/Ts highlights the importance of all themes and related factors for the sustainability of EBPs within acute care contexts. The innovation [40], leadership and management [34, 40, 41, 43], outer context [39–41], and outcome [34, 40, 41, 43] themes were not evident in all F/M/Ts. The *Framework and a Measurement Instrument for Sustainability of Work Practice* (hereafter Slaghuis FMIS-WP) [40] contains only three themes and related factors as it represents a portion of a larger conceptualization on sustainability unpublished. The 37 *core* factors primarily are distributed among 6 themes, given the outcome concept/factors are undefined. All F/M/Ts contain *core* factors from the adopter, inner context, and process themes. Fifty-seven percent (21 out of 37) of the *core factors* are contextual contingent including inner context, inner processes, and outer context core factors thus highlighting the influence context may have on the sustainability of EBPs in acute care. One F/M/T contained all 16 *common* factors [13].

#### Concept/factors relationships

All originators described the relationship between the factors as non-discrete or dynamic, which may interact either in varied combinations or degrees on different levels. How this occurs, however, was not made explicit by definition/statements. The use of arrows to imply direction or potential influence between concepts/factors was used in seven F/M/Ts. Uniquely, the *National Health Service Sustainability Model* (hereafter Maher NHS-SM) [39] originators used three overlapping colored circles representing broad concepts to illustrate a level of dynamic interaction among the related factors within the concepts. The use of arrows or circles failed to clarify how the interactions between factors occurred. The *Dynamic Sustainability Framework* (hereafter Chamber DSF) [34] originators specified a “dynamic relationship” that exists between and among the three concepts (e.g., innovation, practice setting, broader system) and changes over time, but how to interpret this was unclear. Uniquely, the *DCOM Framework with Realistic Evaluation* (hereafter Frykmann DCOMF) [41] originators used relational statements to identify key influences impacting relationships between factors not evident in other F/M/Ts, namely, four mechanisms of behavior change: direction, competence, opportunity, and motivation. All originators recommended further testing to seek greater clarity about relationships between concepts. Fleiszer SHIF [13] originators suggest their framework is representative of a mid-range theory, and further understanding of the relationship between concepts and factors is essential.

#### Assumptions

Key assumptions underlying the F/M/Ts include (i) the concept of sustainability is only partially mature [13], dynamic [34, 40], or ambiguous having different meanings in different contexts [25]; (ii) sustainability considers change (either strategic and/or incremental) as a central influence [25, 34, 39–41, 43]; (iii) evolving fit and/or adaption of the EBP is expected [13, 25, 34, 39, 40]; and (iv) success overtime is based on whether or not the EBP remains beneficial [13, 25, 34, 39, 41, 42].

#### Schematics

All originators provided schematic representations illustrating key concepts/factors claiming to be operational and able to guide sustainability efforts and future research. Four F/M/Ts depict unidirectional graphical representations that assume a continuum or processual stance focusing on the EBP and its ongoing implementation process in context influenced by internal or external factors [13, 25, 34, 41] thus implying the goal of maximizing the fit between the EBP and the context. Originators of the remaining schematics provided a simple, high-level representation depicting the interplay among the set of factors [39, 40, 42, 43]. Notably, in all schematics, each factor category was represented as equal relative to one another given their image size. In fact, originators contend the relative significance of the factors cannot be determined a priori, except the Maher NHS-SM [39] where relative weighting within and among the factors is provided based on empirical evidence.

#### Empirical testability

To date, evidence of further testing of four F/M/Ts has occurred [13, 34, 39, 40]. Notably, the Maher NHS-SM [39] has been empirically tested in the USA, Canada, UK, South Africa [14, 21, 45, 46] and in low- to middle-income countries [47]. All originators recommended practical testing (application and evaluation) in multiple contexts using different methodologies to broaden conceptual understanding and further development/refinement. Specifically, research using a systemic and process-orientated lens to uncover the complexities and dynamics of the concept was recommended [13].

#### Parsimonious and language clarity

Five F/M/Ts were subjectively rated by coauthors (LNP, BD, IG, CB, JS) as parsimonious, with clear language, terminology, explicit definitions for factors, and without repetitions noted [13, 25, 39, 41, 42]. The remaining three F/M/Ts were rated as partially parsimonious based on the lack of completeness [40] or the use of vague definitions and concept relational statements [34, 43].

#### Logical adequacy

Originators claimed all F/M/Ts as operational and capable of guiding research and practice to explore factors influencing the sustainability of healthcare EBPs. Originators of four F/M/Ts explicitly provided either testable hypotheses [34, 39, 42] or testable scales for the concepts [40]. The chambers DSF [34] originators proposed seven tenets related to the ongoing improvement of EBPs emphasizing a “central goal of continuously optimizing the fit between the innovation and the dynamic (changing) delivery context to achieve maximum benefit” [34]. The *Model for Sustaining Innovations* (hereafter Racine MSI) [42] originators provided 12 propositions, which align with three main factor categories (innovation legitimacies, intermediary functions, conditions of local adopters) but assert it does not predict the likelihood of sustainability. Conversely, the Maher NHS-SM [39] originators defined 10 measurable factors, which are weighted within and among each other, providing a testable hypothesis and a prediction of sustainability for the improvement. originators of the four remaining F/M/Ts [13, 25, 41, 43] identified measurable factors/variables to guide research and data collection without explicitly defining the impact of the factors for outcomes concept but rather state it can vary based on the innovation, conditions, and contexts.

#### Logical fallacies

Minimal inconsistencies related to the content within the “adopters” and “outcomes” themes were noted among all F/M/Ts by coauthors (LNP, BD, IG, CB, JS). Specifically, within three F/M/Ts, the “adopters” theme was not identified as distinct but rather considered part of the inner context [13, 34] or inner processes [41] themes. In the Fleiszer SHIF [13], the deliberate positioning of individual characteristics within the inner context versus distinct, similar to the leadership and management theme, was not explicit. Chambers DSF [34] originators did not identify “staff” separately but rather part of the “practice setting” or inner context. The staff/team member is not explicitly identified as a separate theme by originators of Frykman DCOM [41], yet the entire framework is focused on revealing how behavior change interventions influence the sustainability of staff/teamwork behaviors. The failure to distinguish adopters, either as individual [2, 48] or collective agency [49] influences, as a separate theme by originators is inconsistent with other F/M/Ts noted in recent syntheses [10, 29] and this study. Furthermore, originators of four F/M/Ts, identified “outcomes” as a theme [13, 25, 34, 41] represented by the combined influence of factors from within their frameworks. Outcome factors were undefined in all F/M/Ts.

#### Usefulness

Originators claimed the F/M/Ts have multidisciplinary relevance and practicality to inform health professionals, administrators, policy-makers, and/or funders to identify inadequacies, refine theory, and ensure the development of the concept. Uniquely, originators of Racine MSI [42] contend their model provides a “blueprint or agenda” [42] with clear practical implications. Maher NHS-SM [39] originators assert their model is intended to provide a platform for quality improvement for all healthcare disciplines. Other originators indicated their F/M/T can be used across multiple healthcare settings [34, 41], for nursing-specific settings [13, 43] or at the micro-level of work practice [40]. Originators of *Sustaining Organizational Change Framework* (hereafter Buchanan’s SOCF) [25] presented a practical guide outlining a range of potential influences/factors at different levels of analysis.

#### Tools

Two F/M/Ts provided tools [39, 40]. Maher NHS-SM [39] includes a manual, user guide, diagnostic tools, videos, and an interactive option, all of which can be used to assess and predict the likelihood of the sustainability of change in clinical practice using a systematic approach. The Slaghuis FMIS-WP [40] includes an instrument to measure sustained changed work practices related to improvement processes, which originators have tested [40, 50].

#### Generalizability

The Slaghuis FMIS-WP [40], Fleiszer SHIF [13], and Frykman DCOMF [41] were all designed to guide practice and research in acute care settings. Specifically, Slaghuis FMIS-WP [40] and Frykman DCOMF [41] were designed for changing complex healthcare environments (hospitals) where high turnover and interdependence between multiple professionals often exists. The Fleiszer SHIF [13] was designed for use in diverse hospital nursing contexts at the unit/organizational level. The Fox SITF [43] was recommended for use in unspecified nursing contexts. The Maher NHS-SM [39] was designed to guide practice and research at the project/initiative level and has been tested in several non-specified healthcare settings [21] including hospitals [51], community settings [14], and globally [47]. The Buchanan SOCF [25], Racine MSI [42], and Chambers DSF [34] were designed for use in non-specified healthcare contexts for a broad range of interventions at a project/initiative level [34] or unit/organizational level [25, 42].

## Discussion

This systematic review is the first to include a comprehensive analysis of healthcare sustainability F/M/Ts with a primary focus on identifying key concepts influencing the sustained use of EBPs in acute care contexts. Our search revealed the vast majority of F/M/Ts relating to sustainability were designed specifically for use in community and public health settings, which is congruent with the current literature [3, 10]. Notably, only three F/M/Ts were primarily focused on the sustainability of EBPs within acute care settings [13, 40, 41], and five were recommended for use in non-specified healthcare organizational/settings [25, 34, 39, 42, 43]. Recommended target domains for use across disciplines imply general learning can be gathered to inform sustainability for practice and research using an interdisciplinary approach. Addressing sustainability challenges from a variety of theoretical perspectives and disciplines is equally pivotal to understanding this concept in acute care as reported in other healthcare sectors [3].

The two most commonly described constructs cited in the F/M/Ts for sustainability were

“continued delivery or use” and “evolution or adaption” constructs. The prominence of these constructs emphasizes the continuous use and evolutionary nature of sustained EBPs in context over time and is congruent with Moore et al.’s [1] previous developed definition of sustainability. Furthermore, this analysis provides insight into sustainability as a “process” or “stage/phase” of ongoing/continuous use of EBPs post-implementation. This finding is congruent with researchers who argue sustainability is not an all or nothing “phase or endgame” [34] nor an “outcome” [52] but rather a “*process* of managing and supporting the evolving EBP” overtime [34]. Some contend it is a “matter of degree of sustained change” [18, 53] to be viewed as a “continuous phase” [54] or a “continuum” [14] or a “*process*” [10]. The importance of this construct is consistent with a recent review [10], ultimately adding new knowledge to the current definition [1]. The shift in perspective of sustainability as a “process or ongoing/continuous stage/phase” [3, 10], together with the EBPs’ evolutionary nature and dynamic interaction/influence among the factors overtime [3, 34], highlights the complexity of planning and measuring sustainability and the need to consider how strategies for sustainment overtime differ from implementation and/or potentially overlap.

Results provide a resource of eight F/M/Ts and hypothesized factors that can be used by acute care team members planning or currently implementing EBPs with the goal of improving patient outcomes. Our synthesis of the concepts/factors revealed 37 *core factors* which cluster around 7 *themes specifically defined by the F/M/T originators to be relevant to acute care settings.* Four F/M/Ts containing all [13, 25, 34] or most [42] of the themes provide a knowledge base for practitioners and researchers to evaluate the sustained use of EBPs within their acute care setting. Four themes align with those deemed useful in any setting by Lennox et al. [10] (e.g., (i) initiative design and delivery = *inner processes*, (ii) people involved = *adopters*, (iii) organizational setting = *inner context*, (iv) external environment = *outer context*), and three add to the current knowledge, namely, *leadership and management*, *characteristics of the innovation*, and *outcomes.* The equal distribution of *core factors* among six of the seven themes (excluding outcome) signifies the relative importance of each theme for the sustainability of EBPs in acute care. Notably, several factors support the conceptualization of sustainability as “a dynamic construct that allows for adaptation in response to new or changing populations, evidence, policies, or other contextual influences” [3]. The combined contextual factors (57% or 21/37) influencing sustainability related to acute care contexts include (i) the integration of *four layers of context factors* influencing the sustained use of complex organizational change practices (e.g., individual, interpersonal relationships, internal context, wider infrastructure system) [41]; (ii) *attention to the complexity*, *multi-layered*, *ever-changing* organizational setting [13, 25, 34]; (iii) *the adaptability of the innovation/EBP* to context [13, 34, 39], and (iv) the *dynamic process of routinization of innovations/EBPs* as a source of change [40]. Arguably, contextual factors impacting sustainability within and among departments or sites will likely provide insight into why the sustained use of EBPs may vary within the same acute care setting. In turn, this likely will affect the strategies needed for sustainment.

Differences amongst F/M/Ts lie in the overall structures, the degree of refinement, substantiation to date, and identified gaps. Each F/M/T reflects a different conceptualization of sustainability evident in the varied schematics. The use of vague/minimal terminology defining concepts/factors and their relationships increases the potential for multiple interpretations. Sustainability outcomes were depicted in three F/M/Ts as a range (e.g., decay to sustainability to development [25], a spectrum from high to nil [13] or as an ongoing stage/phase of implementation [41]). The Chambers DSF [34] defined outcomes as the “continuation of intended benefits.” The outcome theme is not explicitly defined in the remaining four F/M/Ts [39, 40, 42, 43]. Consistent with other researchers [3], we recommend future inquiry focus on articulating sustainability outcomes.

Identified gaps among the eight F/M/Ts were revealed by examining their concepts/factors and tools. Variation existed related to the inclusiveness of each factor and labeling of themes. For example, the absence of any type of ‘financial factor’ in the Racine MSI [42] to guide stakeholders offers little insight into how this factor influences sustainability. Additionally, Fleiszer SIHF [13] did not include a separate “adopter theme” but instead recognized the role of leadership and management as distinct. A lack of focus on facilitation as a factor either explicitly or implicitly or its inclusion and the perceived need for it is not evident in most F/M/Ts [25, 34, 39, 41, 42], except the Fleiszer SIHF [13]. Originators of the Chambers DSF [34] were distinct in their acknowledgement of the “dynamic relationship” between three “changing” concepts (innovation, practice setting, broader system), their focus on “benefits beyond helping patients” and the “fit of the innovation” with existing routines/processes. Despite this acknowledgement, the potential risk of overlooking the impact on patient outcomes has been recognized [21]. Slaghuis FMIS-WP [40] originators claim their framework is part of a larger unpublished framework. Lastly, only two of the F/M/Ts included tools to measure changed work practices [39 40]. To date, minimal evidence for instrument reliability and validity is available for these tools.

### Strengths

This systematic review is the first to include a comprehensive analysis of healthcare sustainability F/M/Ts for EBPs with a primary focus on acute care context. Seven themes primarily related to acute care were identified, four that align with a current review [10], and three that add to current knowledge (e.g., characteristics of the innovation, leadership and management, and outcomes). By identifying factors and themes/constructs relevant to acute care settings, this work has the potential to aid sustainability for those planning or currently implementing EBPs. The analysis offers insight into sustainability as a “process” or “ongoing stage of implementation” adding to the current definition. For the first time, factors (mechanisms) influencing the sustainability of behavior changes in an acute care setting (see Table 5) are integrated into a synthesis adding to the current knowledge base [41]. Additionally, the modified theory analysis criteria can be used as a tool to guide practitioners, researchers, and students in the appraisal of emerging or existing F/M/Ts, related concepts, and factors.

### Limitations

There are limitations to consider when interpreting the results of this review. First, a systematic review was conducted for conceptual F/M/Ts related to the sustainability of healthcare EBPs from January 1, 2015, to July 3, 2018. Frameworks/models/theories prior to these dates were identified from two existing knowledge synthesis, dated 1946 to March 2017 inclusively. Inclusion criteria varied within each synthesis, and therefore, there is a risk some F/M/Ts may have been missed. Second, the new systematic review, designed to identify recently published F/M/Ts included four key databases, known to focus on healthcare and/or implementation science, among the 14 combined databases used within the two syntheses. There could be sustainability F/M/Ts in databases restricted to the social sciences or organizational management literature that may have been missed. However, healthcare was the primary focus. Third, the qualitative analysis of the main themes and related factors was conducted independently by one reviewer, then analyzed/reviewed by coauthors. Analysis using a deductive approach might draw different conclusions. Lastly, interpretations made as part of the theory analysis are based on the reviewers’ subjective appraisal [36]. These items are clearly marked in Table 2.

## Conclusion

Sustainability is an emerging field of study. Given the ever-changing nature and complexity of acute healthcare settings and related costs, it is imperative practitioners and researchers consider the use of sustainability F/M/Ts to guide their practice and inquiry to ensure EBPs are sustained effectively, continue to inform clinical decisions and contribute to improved patient outcomes. Principally, selecting one of the eight sustainability F/M/Ts proactively to plan, evaluate and interpret findings is recommended. Then consider the context level for F/M/T use, specify the goals of sustainability, and determine if the concepts and factors listed apply [55]. We also recommend future inquiry adopt the use of mixed methodologies to explore the complex relationship between implementation factors and outcomes (including sustainability), and determine their level of influence using Proctor’s Framework [56]. Additionally, using a theory analysis approach to examine F/M/Ts containing both implementation and sustainability could provide new insight into the relationship of factors over time (e.g., early, mid-process, and long-term) and/or the potential impact of implementation on the sustainability phase.

## Supplementary information

**Additional file 1.** PRISMA 2009 Checklist—Identifying relevant concepts and factors for the sustainability of evidence-based practices within acute care contexts: A systematic review and theory analysis of selected sustainability frameworks.

**Additional file 2.** Concept key terms and search strategy.

**Additional file 3.** Exluded files.

**Additional file 4.** Qualitative analysis of concepts and factors for sustainability frameworks/models/theories.

## Data Availability

All data generated or analyzed during this study are included in this published article (and its supplementary information files).

## Abbreviation

Buchanan SOCF: Buchanan Sustaining Organizational Change Framework
Chamber DSF: Chamber Dynamic Sustainability Framework
EBP: Evidence-based practices
F/M/T: Framework/Model/Theory
Fleiszer SHIF: Fleiszer Sustainability of Healthcare Innovation Framework
Fox SITF: Fox Sustainability of Innovation Theoretical Framework
Frykman DCOMF: Frykman Direction, Competence, Opportunity and Motivation Framework
Maher NHS-SM: Maher National Health Services Sustainability Model
Racine MIS: Racine Model for Sustaining Innovations in their effectiveness
Slaghuis FMIS-WP: Slaghuis A Framework and a Measurement Instrument for Sustainability of Work Practice in long term care
