# Supplementary material for: Identifying relevant concepts and factors for the sustainability of evidence-based practices within acute care contexts: a systematic review and theory analysis of selected sustainability frameworks
Source: Implement Sci. 2019 Dec 19;14:108. doi: 10.1186/s13012-019-0952-9 (PMC6923954; doi:10.1186/s13012-019-0952-9)
Supplement: Supplementary file 2 — Additional file 2. Concept key terms and search strategy. [file 13012_2019_952_MOESM2_ESM.docx]

**Additional file 2**

**Concept key terms and search strategy**

Concept key terms

| **Concept 1**  **Frameworks/Models/Theories** | **Concept 2**  **Sustainability** | **Concept 3**  **Research use or evidence-based** |
| --- | --- | --- |
| Framework(s) | Sustain* (*includes variable endings) | Research |
| Model(s) | Routinization / Routinisation | Research utilization(s) |
| Theory(ies) | Institutionalization / Institutionalisation | Evidence-based practice(s) or guideline(s) |
|  |  | Practice Guideline(s) |
|  |  | Diffusion of innovation(s) |
|  |  | Organizational innovation(s) |
|  |  | Clinical protocol(s) |
|  |  | Program(s) / Programme(s) |
|  |  | Intervention(s) |

**Search strategies**

**Embase Classic + Embase 1947 to 2017 October 25**


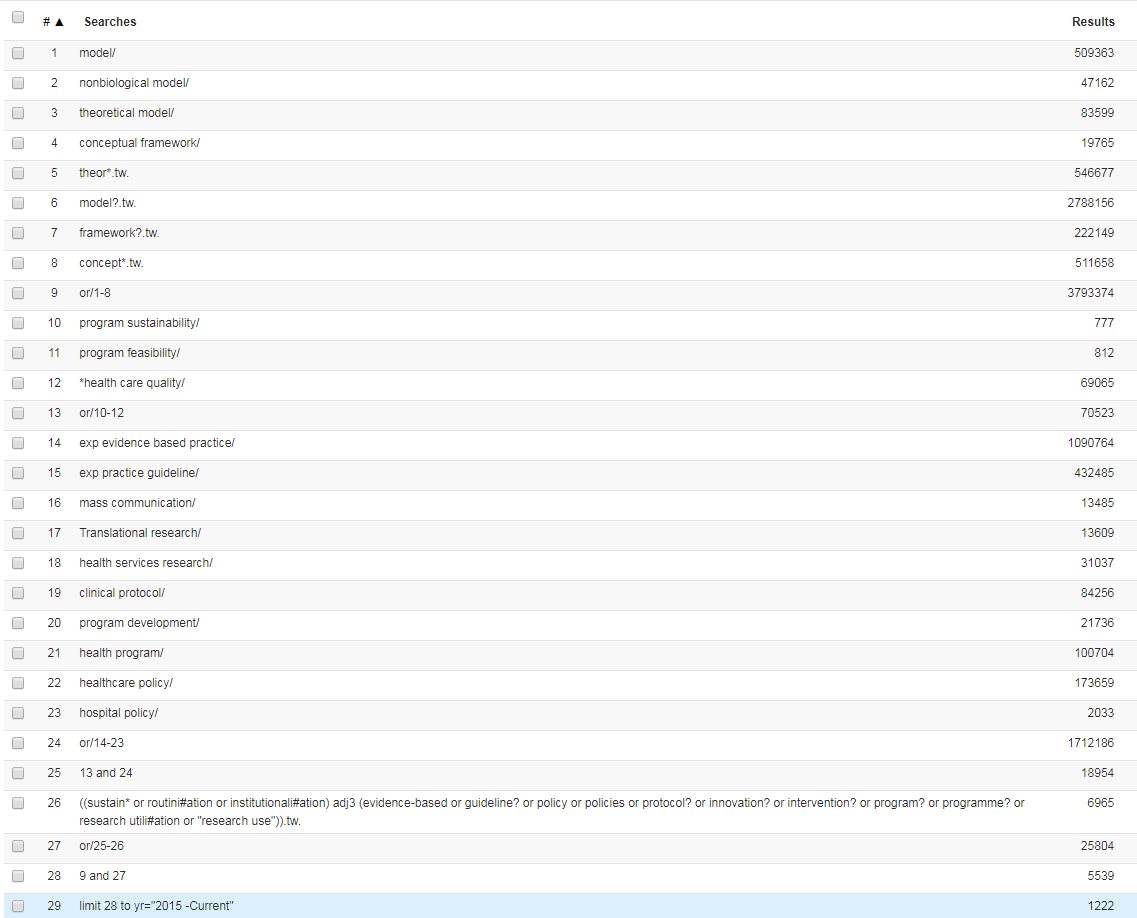


**Ovid MEDLINE(R) Epub Ahead of Print, In-Process & Other Non-Indexed Citations, Ovid MEDLINE(R) Daily and Ovid MEDLINE(R) 1946 to Present**


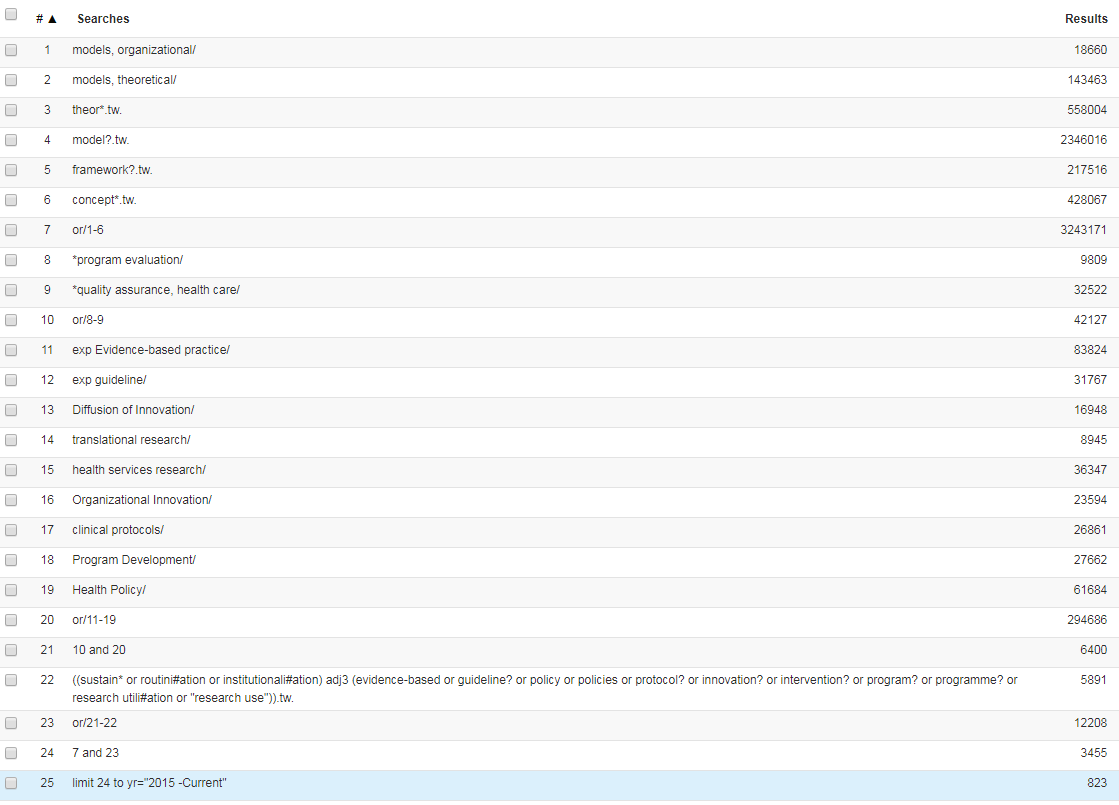


**CINAHL (Ebsco)**

| S1 | (MH "Models, Theoretical") | 26,273 |
| --- | --- | --- |
| S2 | (MH "Organizational Theory") | 513 |
| S3 | (MH "Theory-Practice Relationship") | 2,478 |
| S4 | (MH "Conceptual Framework") | 29,374 |
| S5 | TI model# OR AB model# | 191,696 |
| S6 | TI theor* OR AB theor* | 71,718 |
| S7 | TI framework# OR AB framework# | 42,142 |
| S8 | TI concept# OR AB concept# | 47,307 |
| S9 | S1 OR S2 OR S3 OR S4 OR S5 OR S6 OR S7 OR S8 | 324,912 |
| S10 | (MM "Program Evaluation") | 6,159 |
| S11 | (MM "Quality of Health Care") | 23,071 |
| S12 | S10 OR S11 | 29,137 |
| S13 | (MH "Professional Practice, Evidence-Based+") | 48,564 |
| S14 | (MH "Professional Practice, Research-Based+") | 4,345 |
| S15 | (MH "Health Services Research+") | 14,737 |
| S16 | (MH "Program Implementation") | 15,396 |
| S17 | (MH "Program Development") | 15,943 |
| S18 | (MH "practice guidelines") | 40,880 |
| S19 | (MH "Guideline Adherence") | 5,612 |
| S20 | (MH "Health Policy") | 31,714 |
| S21 | (MH "Diffusion of Innovation") | 7,274 |
| S22 | (MH "Protocols") | 11,218 |
| S23 | S13 OR S14 OR S15 OR S16 OR S17 OR S18 OR S19 OR S20 OR S21 OR S22 | 172,135 |
| S24 | S12 AND S23 | 4,348 |
| S25 | TI ( ((sustain* OR routini?ation OR institutionali?ation) N3 ("evidence-based" OR guideline? OR policy OR policies OR protocol? OR innovation? OR intervention? OR program? OR programme? OR "research utili?ation" OR "research use")) ) OR AB ( ((sustain* OR routini?ation OR institutionali?ation) N3 ("evidence-based" OR guideline? OR policy OR policies OR protocol? OR innovation? OR intervention? OR program? OR programme? OR "research utili?ation" OR "research use")) ) | 1,244 |
| S26 | S24 OR S25 | 5,570 |
| S27 | S9 AND S26 | 1,283 |
| S28 | S9 AND S26 | 262 |

**ProQuest Dissertations & Theses Global**

Search Strategy:

TI,AB(model OR theory OR framework OR concept) AND TI,AB((sustain* OR institutionalization OR routinization) NEAR/3 ("evidence-based" OR guideline OR policy OR protocol OR innovation OR intervention OR program OR "research utilization" OR "research utilisation" OR “research use”)) AND TI,AB(health)

Results= 36

**Other sources**

Rogers (2005). Diffusion of Innovations

Buchanan, Fitzgerald & Ketley (2006). The Sustainability and Spread of Organizational Change

**Follow up search from Oct 1 2017 to July 3, 2018**

| Databases | Platform | Date | # records |
| --- | --- | --- | --- |
| CINAHL | Ebsco | July 2018 | 91 |
| Medline | Ovid | July 2018 | 197 |
| Embase | Ovid | July 2018 | 260 |
| ProQuest Dissertation & Thesis Global‎ | ProQuest | July 2018 | 4 |
| Total of records |  | July 2018 | 552 |
| Total of records after duplicates removed | N=53 | July 2018 | **499** |
